# Ten-year mortality study of dengue in Malaysia

**DOI:** 10.1016/j.ijregi.2024.100455

**Published:** 2024-09-19

**Authors:** Wan-Fei Khaw, Mohd Firdaus Razali, Norliza Shamsuddin, Nur Faraeein Zainal Abidin, Yee Mang Chan

**Affiliations:** Institute for Public Health, National Institutes of Health, Ministry of Health Malaysia, Shah Alam, Selangor, Malaysia.

**Keywords:** Dengue, Mortality, Malaysia, Death rate

## Abstract

**Objectives:**

Understanding the factors that contribute to dengue deaths is crucial for developing effective interventions and improving public health strategies. This study aims to determine the dengue mortality rate in Malaysia from 2012 to 2021.

**Methods:**

All dengue-related death data were obtained from the Department of Statistics Malaysia and were examined from 2012 to 2021.

**Results:**

A total of 1641 dengue-related deaths were identified. The average annual crude mortality rate was 0.5 deaths per 100,000 population, with the highest rate in 2015 at 1.1 deaths per 100,000 population. The central and southern regions had the highest age-adjusted mortality rates. Older age groups had higher mortality rates than younger individuals.

**Conclusions:**

Dengue remains a significant public health challenge in Malaysia. It is essential to prioritize older individuals and urban areas in addressing this issue.

## Highlights

• From 2012 to 2021, Malaysia recorded 1641 dengue deaths, peaking at 325 in 2015.
• The central and southern regions had the highest mortality rates, led by Kuala Lumpur.
• Mortality rates increased significantly with age, highest among those aged 75 years and older.
• Dengue deaths in Malaysia need targeted action for older individuals and urban areas.

## Introduction

In Malaysia, dengue cases have risen dramatically over the past three decades. The incidence rate increased from 44 cases per 100,000 population in 1999 to 399 cases per 100,000 population in 2015. Dengue-related deaths also surged from 45 in 2000 to 134 in 2010 [1]. This trend strains health care services and resources, highlighting the need to understand the factors influencing dengue mortality. Studying these factors can provide insights into the social determinants of health and help develop strategies to prevent the spread of dengue and mitigate its impact. Despite the importance, large-scale studies on dengue mortality are limited. This study aims to examine dengue mortality rates from 2012 to 2021 and identify associated factors.

## Methods

This study is a retrospective analysis of dengue mortality in Malaysia from 2012 to 2021. Data on deaths, including age, sex, and the cause of death with International Classification of Diseases, 10th Revision (ICD-10) codes, were obtained from the Department of Statistics Malaysia (DOSM). Death data were categorized into medically certified deaths, which occur in medical facilities and are certified by physicians, and non-medically certified deaths, which occur outside medical facilities and reported by families to local police. Dengue-related deaths were identified using ICD-10 codes A90 and A91 and DOSM codes 021 and 022. Age was analyzed as a continuous variable and further grouped into six categories: 0-14, 15-29, 30-44, 45-59, 60-74, and 75+ years. Sex was categorized as male or female. The location of death includes 13 states and three federal territories, organized into five regions: central, northern, eastern, western, and east Malaysia. The study included a total of 1641 dengue-related deaths in the analysis; descriptive statistics were performed using IBM SPSS Version 26.0. Crude mortality rates, age-adjusted mortality rates, and mortality rate ratios by age and sex were calculated. Age-adjusted mortality rates were computed using the World Health Organization standard population and mapped using ArcGIS 10.8.

## Results

**Figure 1:**
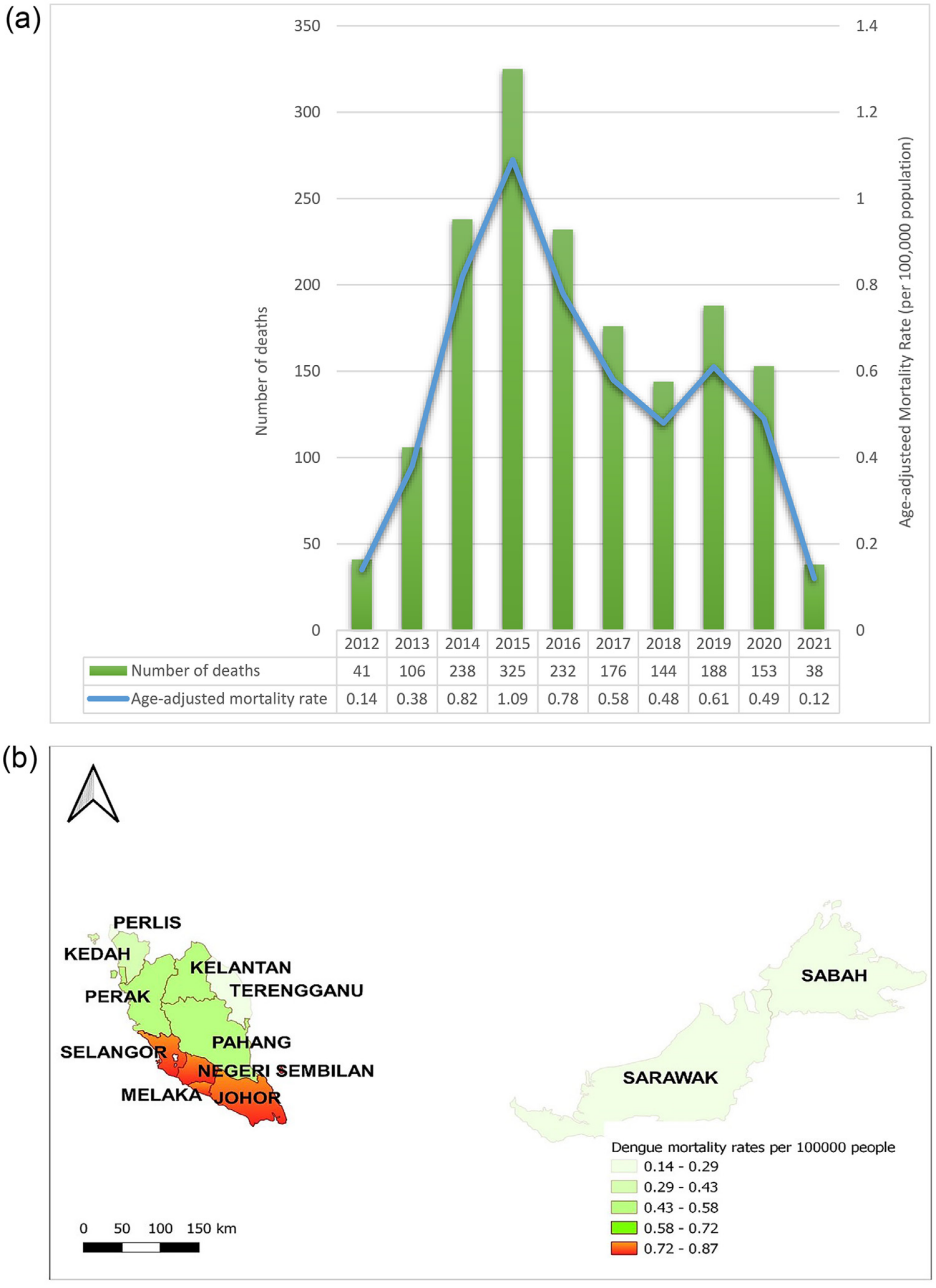
**(a)** Age-adjusted mortality rates (line graph) with numbers of deaths (bar graph) by dengue in Malaysia, 2012-2021. **(b)**. Annual average age-adjusted mortality rates by dengue in Malaysia, 2012-2021.

Between 2012 and 2021 (Figure 1a), 1641 deaths with dengue as the underlying cause were recorded. The average annual number of dengue deaths was 166 (95% confidence interval [CI]: 114.1-218.1), ranging from 38 in 2021 to 325 in 2015. The age-adjusted death rate was 0.1 per 100,000 population in 2012 to 1.1 per 100,000 population in 2015. The rate reduced from 2015 to 2018 and second spike was recorded in 2019 with 0.6 per 100,000 population.

Of the 16 states and federal territories, the central and southern regions had the highest age-adjusted rate and Federal Territory of Kuala Lumpur had the highest age-adjusted rate of 0.89 deaths per 100,000 population, followed by Melaka and Negeri Sembilan with 0.87 deaths per 100,000 population, Johor with 0.86 deaths per 100,000 individuals, and Selangor with 0.85 deaths per 100,000 population (Figure 1b).

Table 1 shows a summary of the characteristics of the cohort. The average annual crude mortality rate was 0.52 deaths per 100,000 population (95% CI: 0.32-0.72), with an age-adjusted rate of 0.56 (95% CI: 0.36-0.76) per 100,000 population. The mean age at death from dengue was 44.4 years (range: 0-106 years). Deaths from dengue were slightly higher in males (50.2%), individuals aged 45-59 years (25.1%), and residents in Selangor (27.5%). These deaths most commonly occurred in hospitals (n = 1563; 95.2%), whereas only 4.8% deaths at home. The average annual age-adjusted rates were almost similar for males and females. The age-adjusted rates increased with age, with 1.64 deaths per 100,000 population in individuals aged older than 74 years. The rates were 10.2 times higher in individuals aged 75 years and above compared to those aged 0-14 years.

**Table 1:** Characteristics of individuals dying of dengue in Malaysia, 2012-2021.

Characteristic	Number of deaths (%)	Crude mortality per 100,000 population per year (95% CI)	Age-adjusted mortality rate per 100,000 population per year (95% CI)	Rate ratio^a^ (95% CI)
**Sex**				
Male	823 (50.2)	0.50 (0.31-0.70)	0.55 (0.36-0.74)	1.00
Female	818 (49.8)	0.54 (0.32-0.76)	0.56 (0.33-0.79)	1.10 (0.96-1.25)
**Age group**				
0-14	150 (9.1)	0.19 (0.11-0.27)	0.19 (0.11-0.27)	1.00
15-29	288 (17.6)	0.31 (0.17-0.46)	0.31 (0.17-0.45)	1.84 (1.10-2.58)
30-44	379 (23.1)	0.55 (0.27-0.82)	0.55 (0.27-0.84)	2.82 (1.95-3.69)
45-59	412 (25.1)	0.88 (0.53-1.24)	0.88 (0.53-1.24)	5.21 (3.50-6.92)
60-74	308 (18.8)	1.29 (0.83-1.75)	1.30 (0.83-1.77)	7.80 (4.94-10.65)
≥75	104 (6.3)	1.63 (1.03-2.23)	1.64 (1.04-2.25)	10.17 (4.65-15.69)
**All**	1641	0.52 (0.32-0.72)	0.56 (0.36-0.76)	

CI, confidence interval.

a Based on crude mortality rates.

## Discussion

The average annual age-adjusted mortality rate stands as 0.56 deaths per 100,000 population. The multifaceted nature of dengue incidence is influenced by climate factors, such as increase in temperatures, humidity, and unpredictable rainfall patterns, which have created favorable conditions for the proliferation of Aedes mosquitoes, the primary vectors for dengue transmission. For example, significant associations between climate variables and dengue incidence were observed in Bangladesh, Dhaka and Chittagong, reinforcing the impact of climate factors on dengue incidence in urban settings [2]. The impacts of urbanization contribute to air and water pollution that can worsen climate change and raise temperatures, leading to higher dengue transmission rates. Warmer temperatures speed up mosquito development, boost their numbers, and increase human contact, resulting in more dengue infections. The link with the rainy season indicates increased transmission of the dengue virus, closely tied to the two monsoon wind periods known as the southeast monsoon (from May to September) and the northeast monsoon (from October to March). Climate variations, such as intense rain, have led to the increase and spread of vectors and intermediate hosts. The presence of breeding sites for mosquitoes, determined by rainfall in an area, can lead to a surge in dengue cases when heavy rain or floods increase mosquito breeding [3]. In addition, humidity and rainfall were identified as critical contributors to dengue transmission, pinpointing rainfall as a significant determinant due to the breeding opportunities it provides for Aedes mosquitoes [4,5].

In 2015, there was a significant spike in dengue incidence. Global warming alters regional and local climates, increasing surface temperatures and shifting local monsoon patterns, which facilitate dengue transmission [6]. Similarly, in 2019, Malaysia experienced a severe dengue outbreak, leading to another notable spike in mortality rates. Dengue epidemics in Malaysia are expected to surge every 4 to 5 years, necessitating preventive control measures to prevent outbreak, such as continuous surveillance and sustained vector control efforts. Public health strategies must focus on reducing mosquito populations and improving community awareness and preparedness to manage and mitigate dengue outbreaks effectively.

In comparison with other regions, the rates of dengue deaths are higher in central and southern of Malaysia. This result was in line with a previous study, which suggested that the highest incidence and death rates were found in central regions, particularly, in Selangor. Furthermore, factors such as population density, urbanization, and health care accessibility contribute to this variation [7]. As a result, giving time and resources to individuals who are most at risk is crucial. In high-risk areas, targeted actions are required to effectively address these variations. These interventions include vector control measures, public awareness campaigns, and strengthening the health care infrastructure.

The older individuals, particularly, those aged 75 years and above, have a greater fatality rate from dengue, possibly as a result of compromised immune systems and underlying medical issues [8]. A previous local study found that increasing age was significantly correlated with severe dengue and dengue-related death [9]. Malaysia has made significant improvements in intensive care and medical technology [10], including the use of high-flow oxygen therapy in intensive care settings and real-time polymerase chain reaction for early detection. These technologies have improved patient outcomes generally; however, because of mobility challenges, underestimating their symptoms, or social isolation, older individuals commonly delay seeking treatment and increase death rates [11]. This emphasizes the importance of early diagnosis, timely treatment, and supportive care tailored to their specific needs. Health care systems need to provide priority to older individuals to effectively address this issue. This includes ensuring older adults have quick access to medical services and offering specialized care designed to meet their specific vulnerabilities.

Males experienced slightly higher dengue mortality rates than females. This finding is consistent with a previous study [9]. Males, often involved in outdoor activities, may be at increased risk in areas where mosquito exposure is higher. Conversely, women and children which spending more time indoors, may face greater risk where indoor mosquito infestation is significant [12]. A previous study found that females of reproductive age tend to develop uncomplicated dengue fever, whereas severe dengue occurs more frequently in males and post–middle-aged females, indicating a biological dimorphism in the response to dengue infection, which may modify the course and outcome of the disease [13]. Furthermore, a previous study highlighted that the delay in seeking treatment among males contribute to higher dengue death rates due to pitfalls in case management, which can result in prolonged shock and multiple organ failures, leading to death [14]. The differences in care-seeking behaviors between genders and psychological factors might be the cause of the death rate discrepancy [8]. Males tend to seek treatment later, which can lead to a higher death rate. Consequently, reducing mortality largely depends on raising public awareness of dengue and promoting early treatment-seeking behavior. In addition, health care providers should be trained to identify and address these gender-based disparities in treatment-seeking behavior, ensuring that all persons receive timely and appropriate care.

It is important to highlight that socio-economic factors may influence how information on dengue mortality is managed in Malaysia. However, the available literature explicitly connecting socio-economic status with dengue mortality are limited. The direct link between socio-economic status and dengue mortality rates also is not clearly established in the research to support that socio-economic factors play a crucial role in shaping dengue mortality and morbidity patterns in Malaysia. In addition, socio-economic factors, such as access to health care, poverty rates, and urbanization, have been implied to play a role in the epidemiology of dengue within Malaysia and in similar dengue-prone regions. For instance, by calculating the likelihood of a vector's presence in a specific area, we can then evaluate how socio-economic, demographic, and climatic variables influence the incidence of dengue. A previous study showed that a higher income usually means lower dengue incidence, related to improved education, better jobs, and better access to health information or medical technology linked to aspects such as care-seeking behavior [15].

Despite a rise in cases, mortality rates have declined due to improved clinical management and timely medical interventions. The Ministry of Health Malaysia has implemented an integrated strategy for dengue prevention and control through the National Dengue Strategies Plan [4]. The mandatory reporting of suspected dengue cases within 24 hours via an e-notification system. This prompt reporting facilitates rapid response through immediate vector control measures such as fogging, enhanced epidemiological surveillance for quick outbreak identification, and increased public awareness, encouraging timely medical attention to reduce complications and mortality. In 2015, the Ministry of Health introduced the rapid combo tests (including nonstructural protein 1 antigen, dengue immunoglobulin M and G antibodies) into the diagnostic tests in addition to the previous dengue definitive diagnosis test which can only be confirmed in the laboratory by dengue serology tests. These rapid combo tests are effective in the initial phase of infection when the virus is present in the bloodstream and when antibodies against dengue start to increase. Therefore, the changes of the laboratory criteria in dengue clinical case definition led to increasing of the reported case, which are compatible with the clinical description and laboratory confirmed which are vital for early detection and confirmation of dengue, enabling prompt interventions to prevent severe dengue and reduce mortality [16].

Moreover, effective locality vector control includes chemical and biological insecticides, community engagement, and regular surveillance to reduce mosquito populations and prevent the spread of dengue fever. Overcoming dengue mortality requires collaboration with various agencies, including local councils, other ministries, and private sectors. This multi-agency approach ensures a comprehensive and coordinated effort, combining resources and expertise to tackle the dengue problem more effectively.

In conclusion, dengue continues to pose a significant public health challenge in Malaysia; however, the high dengue incidence does not necessarily correlate with high mortality rates. Effective vector control and public health initiatives can greatly reduce the spread and mortality of the disease. When tackling this issue, it is imperative to give priority to the older population and urban areas, especially in central and southern of Malaysia. To effectively combat dengue, substantial vector control actions must be implemented along with improvements to health care system and community engagement initiatives.

## Declarations of competing interest

The authors have no competing interests to declare.
